# Automation of *N*-glycan purification using amine-functionalized NiFe_2_O_4_ magnetic nanoparticles

**DOI:** 10.1038/s41598-026-54879-1

**Published:** 2026-06-08

**Authors:** Dalma Dojcsák, Ágnes Mária Ilosvai, László Vanyorek, Csaba Váradi

**Affiliations:** 1 Higher Education and Industrial Cooperation Centre, University of Miskolc, Miskolc, 3515 Hungary; 2 Institute of Chemistry, University of Miskolc, Miskolc, 3515 Hungary

**Keywords:** NiFe_2_O_4_-magnetic nanoparticles, N-glycan purification, Automated sample preparation, Liquid chromatography, IgG, Total serum *N*-glycosylation, Biochemistry, Biological techniques, Biotechnology, Chemistry, Nanoscience and technology

## Abstract

Magnetic nanoparticles (MNPs) are increasingly utilized in biomedical applications, including nucleic acid isolation, protein purification and glycan enrichment, owing to their high surface-to-volume ratio, tuneable surface chemistry and compatibility with automation. Here, we report the development, systematic optimization and automated implementation of an amine-functionalized NiFe_2_O_4_-based MNP protocol for *N*-glycan purification. Critical workflow steps, including particle drying and dispersion, binding efficiency, and elution buffer selection, were comprehensively evaluated. The optimized protocol, employing 0.5 mg/mL NiFe_2_O_4_-NH_2_ particles and 150 mM ammonium-formate (pH 4.4) as elution buffer enabled the reproducible identification of the major *N*-glycan peaks from human serum and immunoglobulin G (IgG). Importantly, the method was successfully translated to a Hamilton Microlab Prep robotic liquid handling platform in a 96-well format, ensuring scalability and reduced manual variability. Overall, this study advances a previously established MNP-based glycan purification approach into a robust, scalable, and automation-compatible workflow through synthesis redesign, systematic optimization, and successful robotic implementation.

**Supplementary Information:**

The online version contains supplementary material available at 10.1038/s41598-026-54879-1.

## Introduction

*N*-glycosylation is a key post-translational protein modification with high relevance in diagnostics and disease monitoring, furthermore it holds great potential to form basis for future personalized therapies. For the analysis of glycoconjugates, analytical methods, such as capillary electrophoresis (CE), ultra-high performance liquid chromatography (UHPLC) and mass spectrometry (MS) or often their combinations, provide high sensitivity and resolution^1–3^. Nevertheless, the major bottleneck remains sample preparation, which should ideally be reproducible, high-throughput and both cost-and time-efficient. Modern hydrophilic interaction liquid chromatography (HILIC)-based techniques are widely applied in both sample preparation – such as functionalized solid-phase extraction (SPE) techniques – and in analysis, for instance through HILIC-UHPLC method^4^. The analysis of *N*-glycans by HILIC-UHPLC requires a fluorescent derivatization to enable the determination by fluorescence detection (FLD). The analysis of the *N*-glycans requires efficient removal of proteins, salts and excess fluorescent labels. Conventional sample preparation approaches – including vacuum centrifugation, SPE columns – are often labour-intensive, expensive or time-consuming techniques. More recently, advanced alternatives such as SPE 96-well plates, filter tips and magnetic bead-based methods, aiming to support the large-scale workflows^5–7^.

Magnetic nanoparticles (MNPs), are already widely applied in isolation of micro- and macromolecules, including nucleic acids, protein isolation and represent a promising alternative for glycan purification through their hydrophilic surfaces functionalized with specific groups^8–10^. Their rapid handling via pipetting, elimination of centrifugation or filtration steps makes the method highly suitable for automation and offers high potential in glycomics^11^. Importantly, their cost-effectiveness has also been demonstrated in a recent study where MNP-based DNA isolation compared with commercial kits^12^. Although MNPs have been applied for glycan adsorption, systematic optimization of dispersion media, binding and elution conditions remained unlimited. To date, the integration of MNP-based glycan purification into fully automated liquid handling workflows remains largely unexplored^13,14^.

Here, we present the further development and optimization of a MNP-based *N*-glycan purification protocol using NiFe_2_O_4_-NH_2_ nanoparticles to ensure colloidal stability and homogeneous particle distribution. To this end, a new batch of MNPs was synthesized through a redesigned synthesis pathway, building on the previously reported co-precipitation method. While, the previously developed protocol demonstrated that NiFe_2_O_4_-NH_2_ MNPs are promising candidates for glycan adsorption^15^; this re-synthesis and rational redesign were necessary to achieve compatibility with the automated liquid handling system. By omitting the drying step of the previously applied co-precipitation synthesis protocol, two types of dispersion media were investigated at varying concentrations, to reduce aggregation and sedimentation, which adversely affected the pipetting steps. In addition, this approach encompassed the elimination of the aqueous and/or alcoholic washing procedures, resulting a more sustainable method by the waste reduction. The aim of the redesigned protocol was the direct utilization of the MNPs without intermediate processing steps to enhance the overall environmental consciousness of the protocol. For comparison with commercially available column-based kits, the elution buffer was also evaluated. The previously applied Milli-Q water elution was assessed with the buffered solution supplied with the kit, which was anticipated to be suitable for assessing separation efficiency as well. Finally, we demonstrate the successful implementation of the optimized protocol and modified dispersion medium of NiFe_2_O_4_-NH_2_ MNPs on a Hamilton Microlab Prep liquid handling robotic platform, enabling reproducible and high-throughput sample preparation for UHPLC-FLD analysis both of serum- and IgG-derived *N*-glycans.

## Results

This study was designed with the dual objective of optimizing a previously developed MNP-based *N*-glycan purification workflow and adapting it for implementation on an automated liquid handling platform. Although the reference MNP-based purification protocol – defined here as a 4 mg/mL suspension of NiFe_2_O_4_-NH_2_ MNP synthesized via a co-precipitation method, followed by drying and subsequent redispersion in water – reproducibly yielded characteristic serum *N*-glycan profiles in a large clinical sample set^15^, further refinement was required to enable the automated implementation.

### Validation of the reference protocol

To establish a reference baseline for all subsequent optimization steps, the previously validated in-house synthetized amine-functionalized ferrite MNP (NiFe_2_O_4_-NH_2_) was re-applied (Protocol A). The overall workflow is consisted of an enzymatic *N*-glycan release by PNGase F digestion, fluorescent labelling by 300 mM procainamide (ProA), MNP-based purification, UHPLC separation, and automated data extraction in Empower 3. This protocol consistently produced typical serum *N*-glycan pattern^3,16^, serving as the baseline for comparison with the optimized dispersion media, elution conditions, and robotic implementation.

### Optimization of dispersion media and concentration

A systematic, multi-parameter evaluation of MNP dispersion media and concentration was performed to improve particle dispersibility and binding efficiency (Table 1). Two dispersion media were tested across a concentration range: glycol (Protocol B) with four different dilutions (30×, 20×, 15×, 10× diluted) and aqueous phase (Protocol C) at six concentration points (16, 8, 4, 2, 1, and 0.5 mg/mL MNP). During evaluation, 41 distinct glycan peaks extracted and provided the total glycan fluorescence intensities [EU], relative abundance stability in each protocol methods not just for optimization, but validation across orthogonal metrics.

**Table 1:** Overview of the key differences between the applied NiFe_2_O_4_-NH_2_ MNP preparation protocols.

Parameter	Protocol A (reference)	Protocol B (glycol dispersion)	Protocol C (aqueous dispersion)
Washing (aqueous)	Yes	No	Yes
Washing (alcoholic)	Yes	No	No
Drying step	Yes	No	No
Final dispersion media	Water (after re-dispersion)	Glycol (as-synthesized)	Water (as-washed)
Particle handling	Re-dispersed after drying	Direct use without processing	Direct use after washing
Expected dispersion stability	Low (aggregation, sedimentation)	High	High
Automation compatibility	Limited	Improved	Improved

Protocol A represents the previously published reference method, involving both aqueous and alcoholic washing steps followed by drying and re-dispersion. Protocol B omits all washing and drying steps, maintaining the particles in the original glycol synthesis medium. Protocol C includes aqueous washing but avoids drying, resulting in direct dispersion in water.

**Fig. 1:**
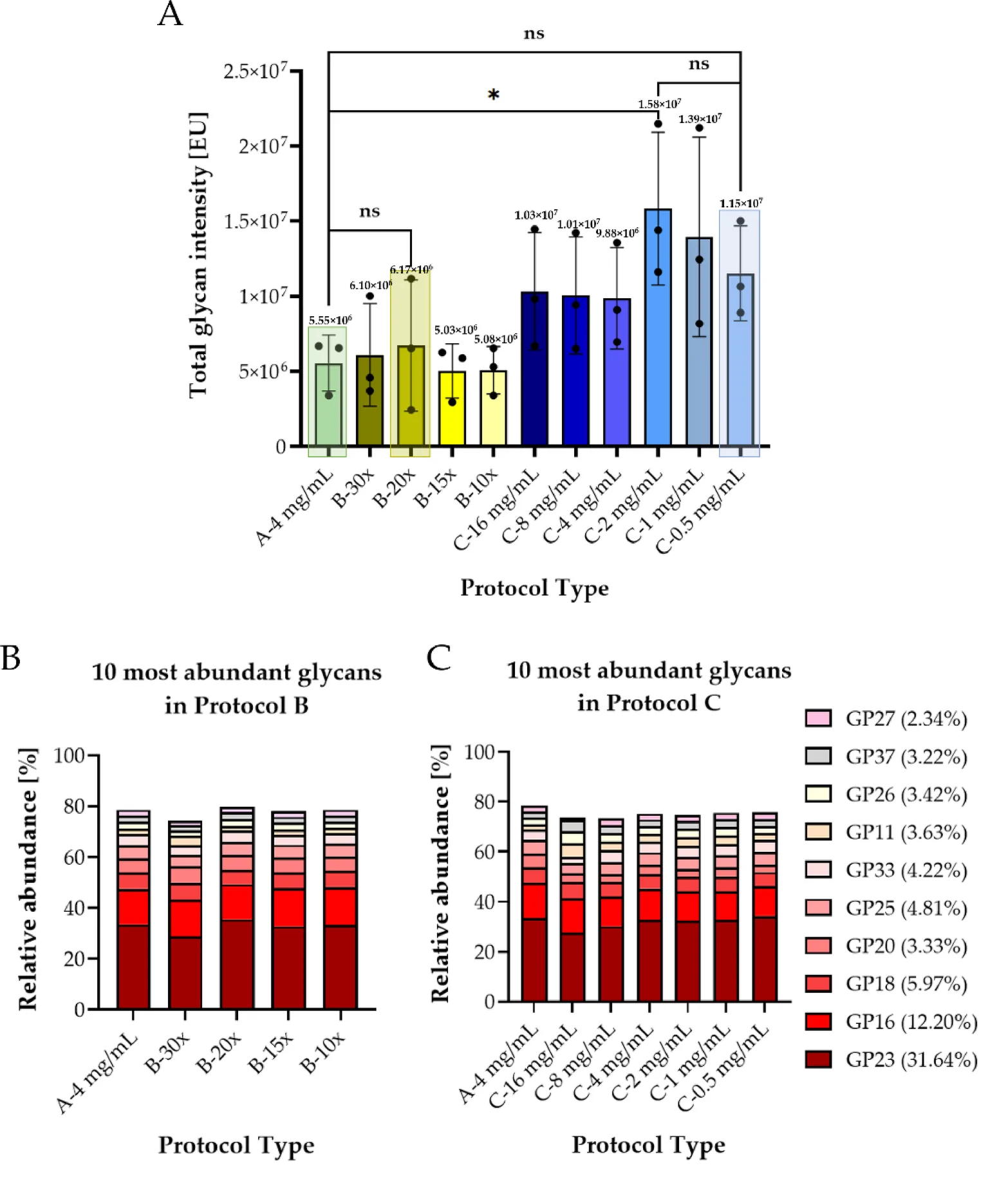
Optimization of MNP dispersion conditions and their impact on fluorescence intensity and glycan distribution. (**A**) Total glycan fluorescence intensities obtained by using different MNP protocols: Protocol A—reference, 4 mg/mL MNP; Protocol B—glycol phase, 30×, 20×, 15×, and 10× dilutions; Protocol C—aqueous phase, 16, 8, 4, 2, 1, and 0.5 mg/mL. (**B**) Relative glycan abundances by depicting the ten most abundant glycoforms across Protocols A-B. (**C**) Relative glycan abundances by depicting the ten most abundant glycoforms across Protocols A-C. Darker colours indicating higher abundance and lighter shades representing lower abundance *N*-glycans. Averaged relative abundances indicated in the brackets. Statistical test results provided in Tables S1, S2.*p < 0.05; ns, not significant.

One-way ANOVA test (Table S1) did not reveal statistically significant differences in total glycan intensities among the tested conditions, with the exception of the comparison between Protocol A and Protocol C-2 mg/mL. Nevertheless, a clear trend was observed, as Protocol C consistently yielded higher total glycan intensities compared to the reference Protocol A, as well as to all tested dilution of Protocol B (Fig. 1A). The relative standard deviation (RSD) values were slightly higher than expected; however, this increased variability can likely attributed to the presence of an outlier among the three parallel measurements. This effect was observed even under conditions associated with high signal intensities. Notably, the lowest RSD value (27.36%) was obtained for Protocol C-0.5 mg/mL (Table S1).

It is important to note that, among the identified *N*-glycans, the distribution of the ten most abundant glycoforms were visualized using stacked bar charts to confirm that their relative abundances remained consistent (Fig. 1B,C). Additionally, the results from Kruskal-Wallis test supported the total relative abundances were not changed significantly (Table S2).

The observations was further supported by the RSD values of the relative abundances, which remained below ~ 20%, with the exception of a few lower-abundance glycoforms, thereby confirming the reproducibility of the methods (Table 2).

**Table 2:** Descriptive statistics of the relative distributions (RA%) of the ten most abundant glycoforms obtained using Protocols B and C, including the corresponding control values.

	Glycoform (av. RA%)	Number of values	Minimum	Maximum	Range	Mean	Std. deviation	RSD [%]
10 most abundant glycoforms in Protocol B	GP23 (32.65%)	5	28.89	35.53	6.64	32.81	2.43	7.39
	GP16 (14.49%)	5	13.79	15.02	1.23	14.41	0.52	3.58
	GP18 (6.24%)	5	5.71	6.72	1.01	6.24	0.36	5.74
	GP20 (5.91%)	5	5.63	6.33	0.70	5.85	0.28	4.71
	GP25 (4.93%)	5	4.40	5.31	0.91	5.00	0.37	7.38
	GP33 (4.06%)	5	3.85	4.46	0.61	4.12	0.28	6.73
	GP11 (2.46%)	5	1.74	3.84	2.10	2.37	0.84	35.31
	GP26 (2.55%)	5	2.22	2.75	0.53	2.59	0.22	8.40
	GP37 (2.25%)	5	1.88	2.48	0.60	2.29	0.25	10.72
	GP27 (2.06%)	5	1.72	2.26	0.54	2.08	0.21	10.12
10 most abundant glycoforms in Protocol C	GP23 (31.64%)	7	27.50	34.30	6.80	31.91	2.31	7.25
	GP16 (12.20%)	7	11.40	14.07	2.67	12.45	1.11	8.93
	GP18 (5.97%)	7	5.80	6.50	0.70	6.02	0.26	4.29
	GP20 (3.33%)	7	3.00	5.63	2.63	3.63	0.92	25.26
	GP25 (4.81%)	7	4.20	5.31	1.11	4.89	0.35	7.07
	GP33 (4.22%)	7	2.50	4.80	2.30	4.24	0.78	18.40
	GP11 (3.63%)	7	2.02	5.30	3.28	3.40	1.02	29.89
	GP26 (3.42%)	7	2.75	4.80	2.05	3.32	0.70	21.18
	GP37 (3.22%)	7	2.48	4.50	2.02	3.13	0.66	21.06
	GP27 (2.34%)	7	1.20	2.70	1.50	2.31	0.52	22.58

All calculations were performed using GraphPad Prism software (version 10.1.2).

The Fig. 1A clearly demonstrates that lower MNP concentrations in Protocol C resulted in higher fluorescence intensities. Since one of the aims of the optimization was to achieve cost-effectiveness and facilitate robotic adaptation, the lowest tested concentration of MNP in Protocol C was selected (0.5 mg/mL MNP) for further experiments. Even at this low concentration, the mean total glycan fluorescence intensity increased by approximately 50% compared to the 4 mg/mL MNP baseline applied in Protocol A (indicated in red in Table S1). However, the Protocol B-20× was additionally selected to assess whether the effect of the elution buffer is independent of the dispersion medium or not.

### Comparative evaluation of elution buffers and commercial kits

**Fig. 2:**
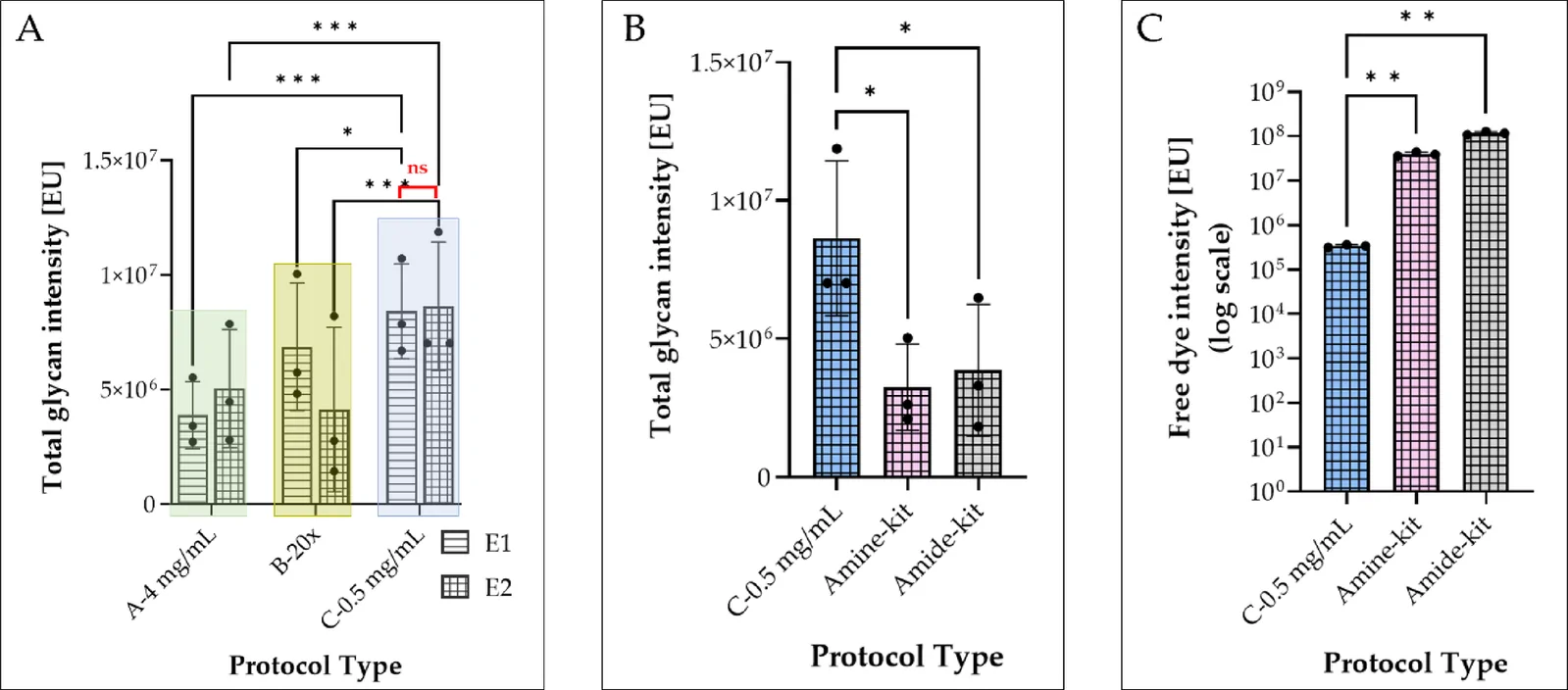
Integration of the MNP-based protocol with different elution buffers and comparison with non-MNP-based glycan purification protocols. (**A**) Comparison of two elution buffers (E1) Milli-Q water; (E2) 150 mM ammonium-formate (pH 4.4) by total glycan intensity. (**B**) Comparison of MNP-based and non-MNP-based methods by glycan recovery and (**C**) free dye intensity. Free dye intensities visualized on logarithmic scale, however the raw chart is provided in Fig. S2. Statistical test results provided in Table S3-S4. *p < 0.05; **p < 0.01; ***p < 0.001; ns, not significant.

In terms of the elution buffers the optimized protocols was carried out with elution by Milli-Q water, as applied previously^17^, and with 150 mM ammonium-formate (pH 4.4), which is derived from the manual of the MonoSpin commercial kit (GL Sciences Inc.). In all cases, 44 distinct glycan peaks were reproducibly identified and evaluated based on total glycan fluorescence intensity, relative abundance and free dye fluorescence intensity (Fig. 2A, Figure S1A). The lowest RSD values of total glycan intensities were obtained by Protocol C-0.5 mg/mL MNP approaches (Table S3). Comparative evaluation of the elution buffers clearly demonstrated that high glycan intensities were achieved with Protocol C, regardless of the buffer used (Table S3). Although no significant differences were observed between the two buffers within Protocol C, 150 mM ammonium-formate (pH 4.4) was selected as the standard elution buffer due to its chromatographic compatibility, ensuring better sample stability during LC injection.

To complete the optimization, Protocol C with the 0.5 mg/mL MNP concentration was directly compared with two commercial spin column kits functionalized with amine or amide groups. The evaluation of total glycan, and free dye fluorescence intensities confirmed that the MNP-based protocol significantly outperformed the commercial counterparts, yielding higher glycan recovery and markedly reduced background. (Fig. 2B,C, Table S3).

When combining data from the different MNP protocols with various elution buffers as well as from the commercial kit experiments, descriptive statistics of the relative abundance of the 10 most abundant glycoforms consistently yielded RSD values below 20%, which further supports the robustness and reliability of the comparative analysis (Figure S1, Table S4).

### Application of the optimized protocol on robotic liquid handling platform

The final optimized protocol employing 0.5 mg/mL NiFe_2_O_4_-NH_2_ MNP (Protocol C) was implemented on a Hamilton Microlab Prep liquid handling platform, which presents several technical challenges, particularly due to the need for stable particle dispersion. Aggregation and sedimentation can compromise pipetting accuracy, leading to variability and reduced reproducibility. Therefore, ensuring homogeneous nanoparticle suspensions was essential for reliable automation. During sample preparation, manual and automated purification workflows were performed parallel. All experiments were conducted in a 96-well plate, in contrast to the earlier experiments carried out in 1.5 mL Eppendorf tubes. Importantly, the use of a 96-well plate format enables the processing of larger sample numbers, providing a more suitable platform for high-throughput clinical research applications. Here, six serum samples were processed in parallel using both the manual (M) and automated (A) platforms, while eight IgG samples were prepared exclusively using the automated platform. Elution was executed using 150 mM ammonium-formate (pH 4.4), selected for its chromatographic compatibility. Data extraction enabled the identification of 21 predominant glycan peaks from serum and 15 individual glycans from IgG.

**Fig. 3:**
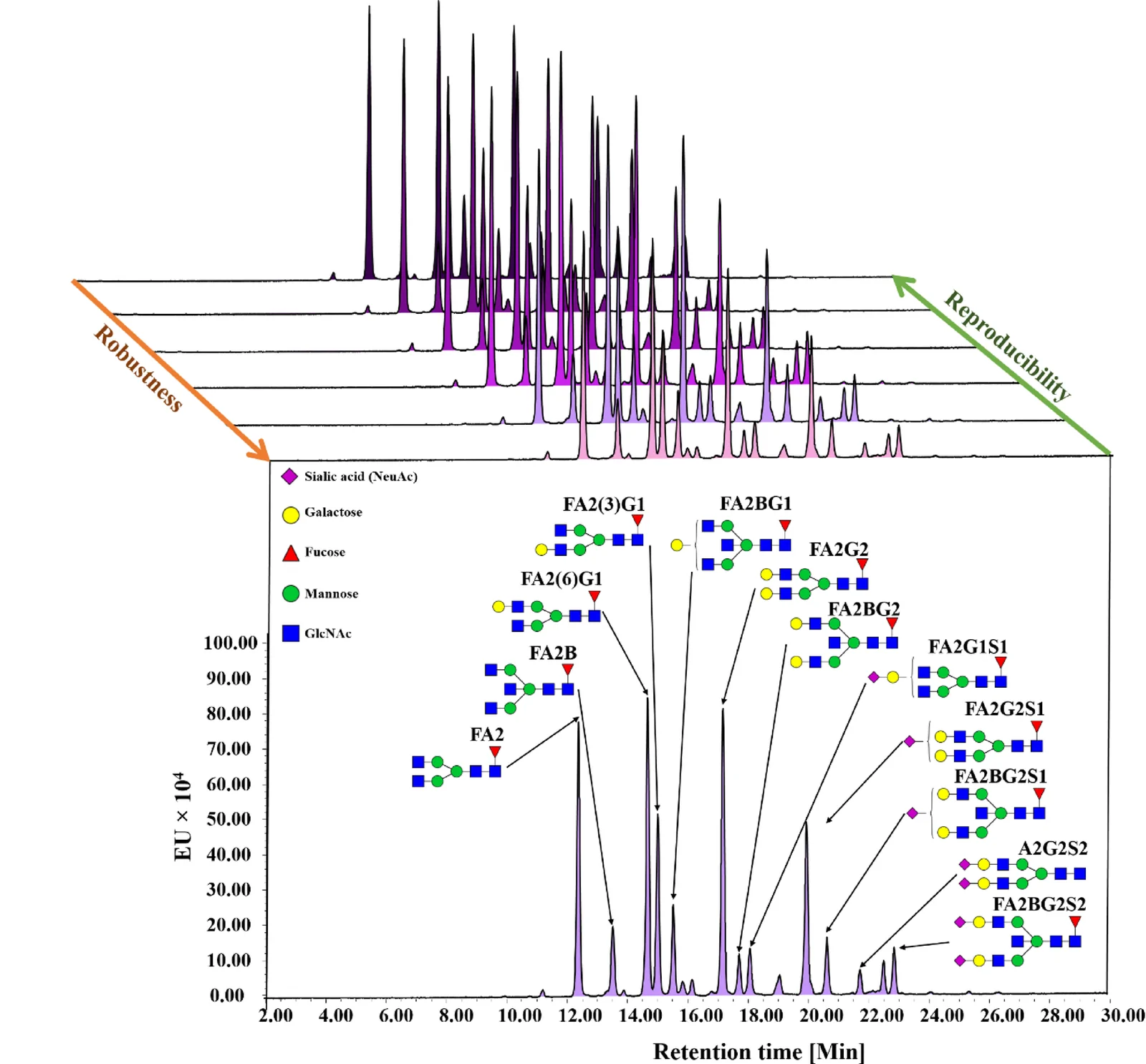
Representative fluorescence chromatograms of six parallel IgG samples processed using the automated MNP-based *N-*glycan purification protocol. Glycan structures are annotated according to the Oxford nomenclature: F, fucose (red triangle); A2, biantennary core structure comprising two GlcNAc (blue squares) and three mannose residues (green circles); B, bisecting GlcNAc (blue square); G, galactose (yellow circles); and S, sialic acid (purple diamonds). Numbers following the G and S symbols indicate the number of galactose and sialic acid residues, respectively. Theoretical m/z^2+^ values are provided in Table S6, Panel E.

Representative fluorescence chromatograms of six parallel IgG samples processed on automated platform are shown in Fig. 3, demonstrating a high degree of overlap and consistent peak patterns across replicates.

**Fig. 4:**
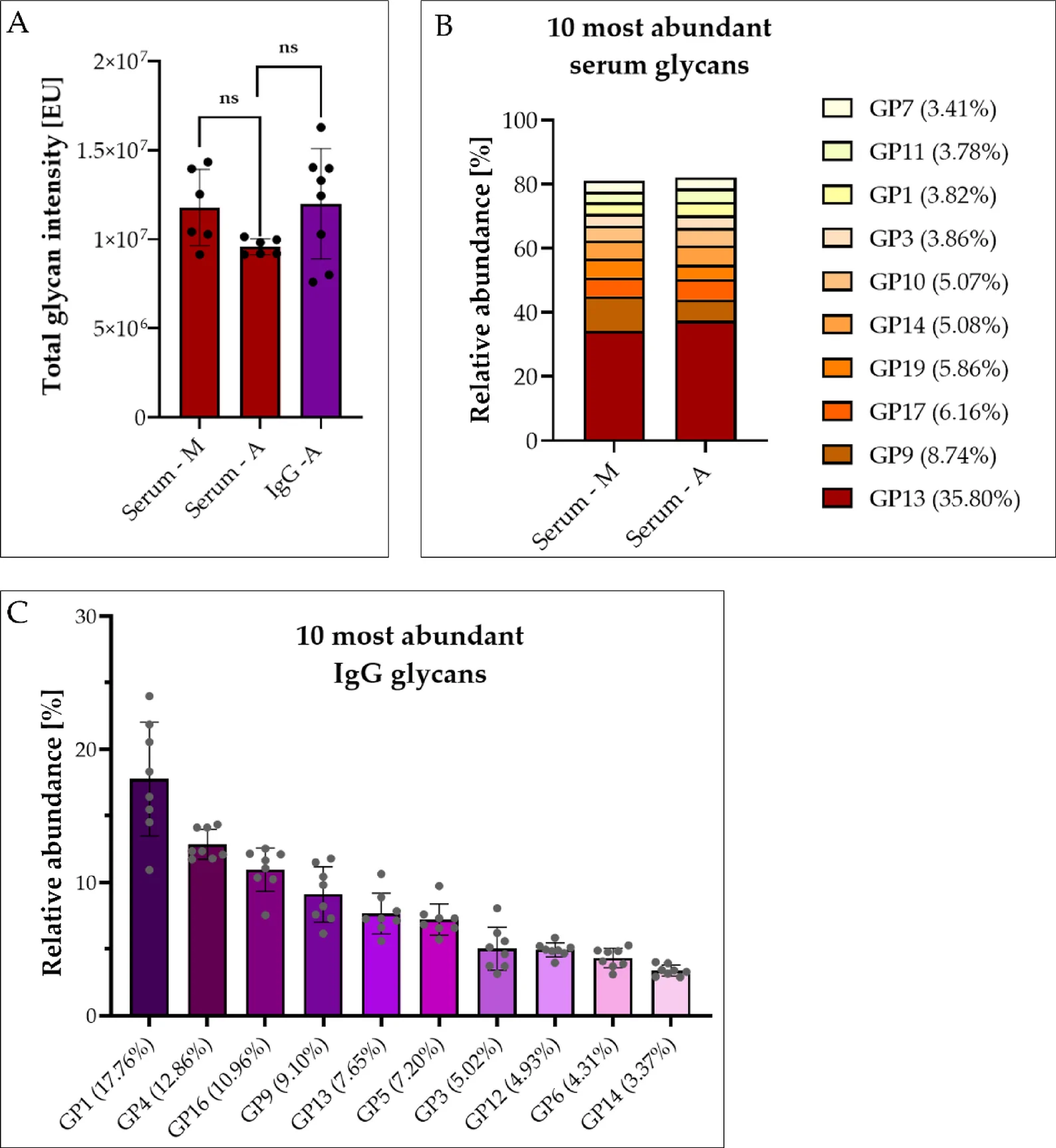
Application of the optimized MNP-based protocol on a robotic liquid handling platform. (**A**) Total glycan intensities obtained from six parallel serum samples showed no significant difference between manual and automated workflows. (**B**) Relative abundances of the ten most prominent serum glycoforms displayed identical distribution patterns across both approaches. (**C**) Relative abundances of the ten most prominent IgG glycoforms in eight replicates. Statistical analyses are summarized in Table S5-S6. ns, not significant.

Figure 4A shows the total glycan intensities obtained from six parallel serum samples, which did not differ significantly between the manual and automated workflows (Table S5). Panel B presents the cumulative relative abundances of the ten most prominent serum glycoforms, which exhibited an identical distribution across both workflows. This observation was further supported by statistical analysis, confirming that no significant changes in glycan distribution were detected (Table S6). The relative distribution of the 10 most abundant glycans from eight parallel IgG samples prepared on the robotic platform, was depicted on Fig. 4C. Although some outliers were observed for the major glycoform (GP1), the less abundant peaks exhibited low variability among replicates, and none showed statistically significant changes (Table S6). The total glycan intensities obtained from IgG samples reached values comparable to those measured in serum, supporting the robustness of the automated MNP-based protocol.

In addition, descriptive analysis demonstrated that the relative peak area distributions of the ten most abundant glycoforms exhibited RSD values as low as 4.7% for serum samples, further confirming the reproducibility of the automated platform (Table S5). In contrast, a somewhat higher variance was observed for IgG GP1 (RSD ~ 24%), which can be attributed to the natural fluctuation in the intensity of the most abundant glycoform peak. Taken together, these values confirm the consistency of the protocol while also reflecting expected biological variation. Overall, results demonstrate that the automated platform faithfully reproduces the performance of the manual protocol with high consistency.

## Discussion

Magnetic nanoparticles (MNPs) have been widely applied in biomedicine since the 1980s, including FDA-approved use as MRI contrast agents and in magnetic particle imaging^18^. MNPs have already been investigated for nucleic acid isolation (from plant cells, human blood and bacterial cells as well), protein purification and glycan enrichment, often demonstrating cost-effectiveness compared with commercial kits^9,19–21^.

Our results indicate that the preparation steps of the MNPs – particularly drying after the MNP synthesis – significantly influence glycan binding efficiency. Dried particles, when re-dispersed in Milli-Q water – as aqueous phase – exhibited poor dispersion stability and caused difficulties in pipetting, which compromises reproducibility (Protocol A). Such limitations may also negatively affect the automated workflows, introducing pipetting errors or contamination risks when executed by robotic arms. In light of these concerns, an alternative approach was designed, omitting the drying step and either keeping the MNPs in a glycol-based dispersion media (Protocol B) or washing them from the glycol and suspending them in aqueous phase media (Protocol C). Both conditions yielded higher total glycan intensities, than the benchmark Protocol A. In addition, the concentration of the MNP was also a critical factor. Increasing particle amounts did not linearly correlate with glycan adsorption property. Instead, optimal glycan recovery was achieved at lower concentrations (2- 1- and 0.5 mg/mL), as demonstrated in Protocol C, which produced the highest glycan signals compared with the Protocol A, besides an equal glycan distribution. This is in agreement with previous reports on magnetic particles, where higher particle concentrations promote aggregation, leading to reduces effective surface area and limited accessibility of binding sites^22,23^. Such aggregation can also introduce mass transfer limitations, further decreasing adsorption efficiency. Importantly, the identification of an optimal low-concentration regime (0.5 mg/mL) demonstrates that glycan recovery is not directly proportional to particle amount. Both of omitting the drying step of the MNP and the dispersion media primarily affected the recovery efficiency rather than glycan composition. Similar effects of drying-induced aggregation and reduced redispersibility have been widely reported for nanomaterials^24^.

Furthermore, the developed protocol is considered environmentally friendly, as the omission of aqueous and alcoholic washing steps did not adversely affect the reproducibility of the glycan-adsorption. The method is both environmentally sustainable, with a reduced environmental footprint, and cost-effective. As demonstrated by the calculation presented in Table S8, the estimated production cost is approximately 1.45 EUR/L, which is estimated to be sufficient for the purification of 5000 serum samples in *N*-glycosylation analysis.

To improve desorption efficiency within the MNP-based protocol, elution buffer comparison (Milli-Q water vs. 150 mM ammonium-formate, pH 4.4) was carried out using Protocol A as the benchmark, with Protocol B-20× and Protocol C-0.5 mg/mL selected for evaluation. Protocol C consistently yielded the highest total glycan intensities alongside the lowest residual free dye signal. Notably, no significant differences were observed among the two elution buffers, suggesting that eluent composition is not a limiting factor for the developed Protocol C. Overall the optimized 0.5 mg/mL NiFe_2_O_4_-NH_2_ MNP-based, called Protocol C, outperformed not only the other MNP variants but also the commercially available MonoSpin column-based kits (amine- and amide-functionalized) according to glycan recovery and dye removal efficiency. The MNP-synthesis modification represents not merely a technical adjustment but a critical step toward making MNP-based glycan purification compatible with automated liquid handling systems.

**Fig. 5:**
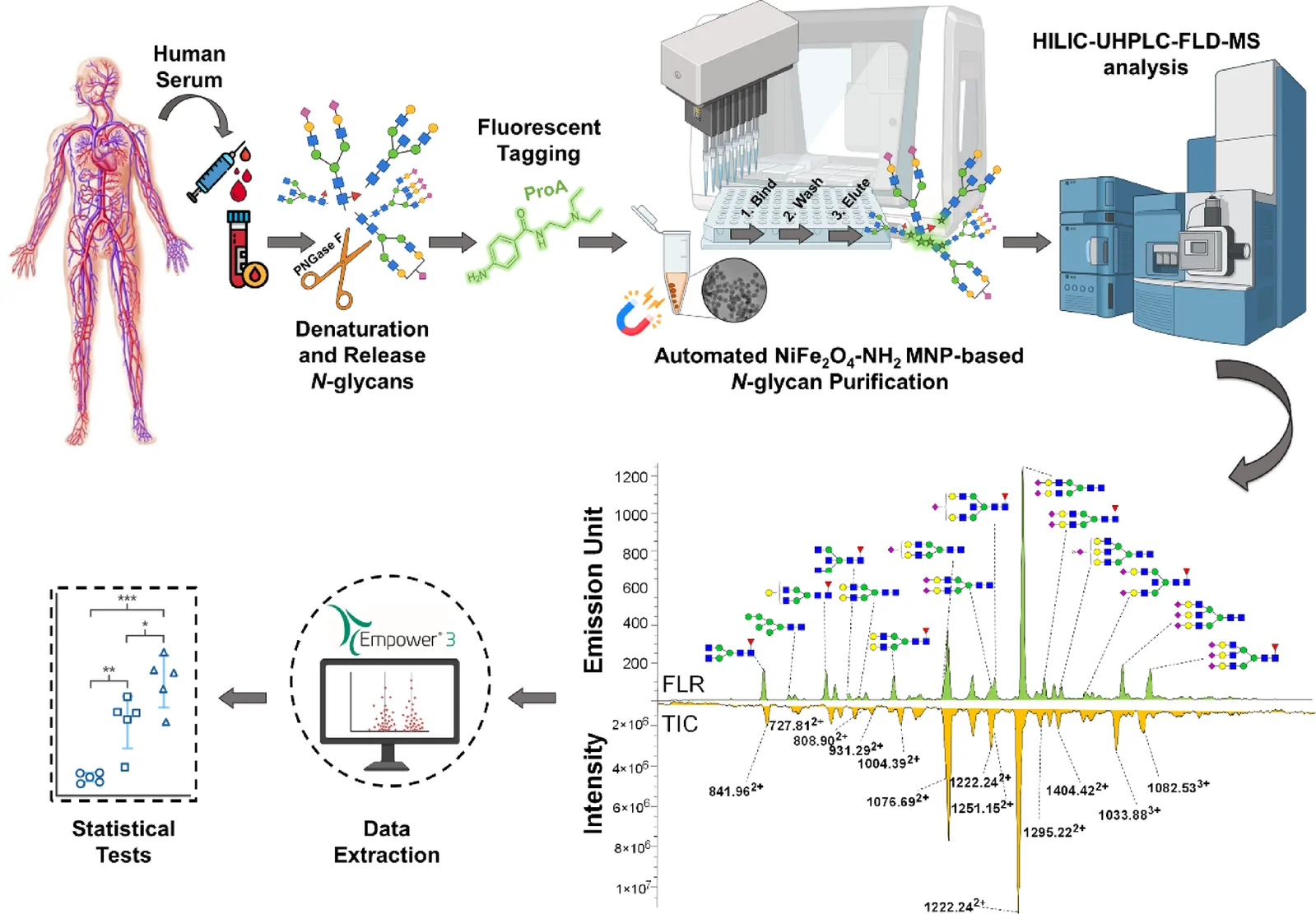
Schematic overview of the applied MNP-based *N*-glycan purification workflow. Human serum sample was denatured, followed by enzymatic release of *N*-glycans using PNGase F and fluorescent labelling with ProA. The tagged samples were purified using NiFe_2_O_4_-NH_2_ MNP-based protocols, including adsorption, washing and elution steps. The sample purification were implemented to robotic platform. Subsequent analysis was performed using HILIC-UHPLC-FLD-MS analysis. Fluorescence chromatograms (FLR) were evaluated by integration of glycan peaks to determine relative abundances, total fluorescence intensities of the *N*-glycans and free dye fluorescence intensity. Total ion chromatograms (TIC) were used for glycan identification based on the corresponding m/z values.

Magnetic nanoparticle-based glycan purification strategies have previously been limited to manual or semi-automated workflows^25,26^. In parallel, automated liquid handling platforms have been successfully applied in high-throughput glycan sample preparation, typically relying on conventional solid-phase approaches^27,28^. In this context, to the best of our knowledge, this is the first study demonstrating the successful application of NiFe_2_O_4_-NH_2_ MNP for *N*-glycan purification on an automated Hamilton Microlab Prep platform using 96-well plate. This scalability is particularly advantageous for large clinical cohorts, where hundreds of samples need to be processed reproducibly in parallel. The reported results ensuring reproducibility and enabling high-throughput serum and IgG derived *N*-glycan purification workflows. The reproducibility of the optimized protocol was further demonstrated by the low RSD values obtained across replicates. These findings highlight MNP-based *N*-glycan purification as a robust, scalable and automatable alternative for glycan sample preparation, with clear potential for integration into routine UHPLC-FLD workflows (Fig. 5).

While the developed nanoparticle-based purification workflow demonstrated robust performance for serum-and IgG-derived *N*-glycans, the present validation was carried out using control serum samples in order to establish methodological reproducibility and analytical robustness under well-defined and biological less variable conditions. This approach allowed us to systematically evaluate the performance of the purification protocol without the confounding effects of disease-associated glycosylation heterogeneity. Nevertheless, the validation on a single-sample type and LC-MS platforms represents a limitation of the study. Furthermore, a potential limitation of the study lies in the batch-to-batch variability inherent to co-precipitation-based nanoparticle synthesis, which may influence particle size distribution, surface functionality, and consequently glycan binding efficiency. Future applications may include high-throughput glycan profiling in autoimmune diseases, oncology, or vaccine response monitoring^29^. As a next step, the long-term stability of MNP suspension should be systematically investigated, however, this was not feasible within the scope of the present study due to time constraints. Finally, future perspectives should include to refine particle surface modifications beyond amine group, explore the application of the MNP protocol for specific glycoprotein (IgG, IgM, acute phase proteins) *N*-glycosylation analysis and broaden the automated workflow for clinical or biopharmaceutical glycomics^14,30^.

## Materials and methods

### Materials

Nickel(II) nitrate hexahydrate and sodium acetate were purchased from Thermo Fisher Scientific (Kandel, Germany). PNGase F enzyme kit was obtained from Asparia Glycomics (San Sebastián, Spain). Borane–pyridine complex (cat. no. 654213) and procainamide hydrochloride (cat. no. P9879) were purchased from Sigma Aldrich (St. Louis, MO, USA). Dimethyl sulfoxide (DMSO, cat. no. 85190) was obtained from Thermo Fisher Scientific (Kandel, Germany). Acetic acid (≥ 99%, LC-MS grade, cat. no. 84874.180), formic acid (99%, LC-MS grade, cat. no. 84865.180), acetonitrile (≥ 99.9%, HiPerSolv CHROMANORM^®^, LC-MS grade, cat. no. 83640.320), and 2-propanol (≥ 99.9%, HiPerSolv CHROMANORM^®^, LC-MS grade, cat. no. 84881.29) were purchased from VWR International (Leuven, Belgium). Ammonia solution (25 v/v%, cat. no. AM02580100) was obtained from Scharlab S.L. (Barcelona, Spain).

### Preparation of glycol (protocol B) and aqueous (protocol C) dispersed NiFe_2_O_4_-NH_2_ MNPs

NiFe_2_O_4_-NH_2_ MNPs were synthesized as previously described^15^, with the modification that the particles were not dried after washing. Instead, two different dispersion conditions were applied:

**(i) Glycol-dispersed MNP (Protocol B)**

Following 12 h reflux synthesis in glycol, the particles were left in the glycol medium without washing. Due to the high boiling point and viscosity of glycol, the absolute concentration could not be determined. Therefore, the initial dispersion was considered as a 1× stock, and serial dilutions (30×, 20×, 15×, 10× diluted) were prepared for testing glycan-binding efficiency.

**(ii) Aqueous MNP (Protocol C)**

After repeated washing with distilled water until clear phase separation was achieved, the particles were kept in the aqueous medium. The concentration of the NiFe_2_O_4_-NH_2_ MNP suspension was determined by drying three 1 mL aliquots on watch glasses at 60 °C for 12 h, weighing the residues, and averaging the results. Based on this, the stock concentration was calculated as 37 mg/mL, and this suspension was used for all further dilutions (16, 8, 4, 2, 1, and 0.5 mg/mL).

### N-glycan sample preparation

For *N*-glycan analysis, serum proteins and isolated IgG from human serum were subjected to enzymatic release of glycans followed by fluorescent labelling and purification.

**(i) Glycan release**

Serum or serum derived IgG samples (9 µL) were denatured at 65 °C for 15 min using denaturation buffer from the Asparia PNGase F kit, resulting in a final volume of 10 µL^29^. After cooling down to room temperature, the reaction mixture was adjusted to 20 µL by adding Milli-Q water, 10× enzyme buffer, and PNGase F enzyme (1 µL). Samples were incubated overnight (O/N) at 37 °C to release *N*-glycans.

**(ii) Fluorescent labelling**

Released *N*-glycans were derivatized with ProA, which had been identified in our previous work as providing superior retention and separation in HILIC-UHPLC-MS system^16^. The labelling solution contained 300 mM procainamide-HCl and 300 mM picoline borane in 70/30 (v/v)% DMSO/acetic acid. 10 µL of labelling solution was added to each sample, and the mixtures were incubated at 65 °C for 3.5 h to ensure efficient fluorescent derivatization.

### Purification of labelled N-glycans

Since fluorescent label was applied in molar excess, glycan samples were purified prior to analytical measurements to remove unreacted dye, serum-derived proteins (including PNGase F), and buffer salts, which could otherwise negatively affect chromatographic separation and MS sensitivity. After derivatization with 300 mM ProA, reactions were quenched by adding 170 µL acetonitrile (ACN), yielding a final volume of 200 µL (85 (v/v)% ACN), an optimal polarity for adsorption of glycans onto amine functionalized surfaces.

**(i) Purification using amine-functionalized magnetic nanoparticles**

1. Conditioning – 200 µL MNP suspension was transferred into 1.5 mL tubes, placed on a magnetic rack for 1 min, and the supernatant discarded.
2. Adsorption – 180 µL of labelled glycan sample (in 85 (v/v)% ACN) was added to the conditioned MNPs, mixed, and incubated for 5 min at room temperature. After an additional 1 min on the magnetic rack, the supernatant was removed and collected. Randomly selected flow-through fractions were analysed by UHPLC-FLD and showed no detectable glycan signal.
3. Washing – To remove non-specifically bound molecules, the MNPs were washed with 200 µL of 95 (v/v)% ACN, followed by 1 min incubation on the magnetic rack and removal of the supernatant.
4. Elution – Glycans were desorbed by suspending the MNPs in 80 µL of elution buffer (Milli-Q water or 150 mM ammonium-formate, pH 4.4 – based on the condition) for 5 min at room temperature. After magnetic separation (1 min), the eluates were collected in clean tubes and used for subsequent analysis.

**(ii) Purification using amine-functionalized MonoSpin Columns**

Commercial amid- and amino-functionalized MonoSpin columns (GL Sciences Inc., Tokyo, Japan) were used both for comparison with the MNP-based method and, in the case of amino columns, for serum *N*-glycosylation studies^30^. The purification procedure followed the manufacturer’s HILIC-based protocol and consisted of four steps.

1. Conditioning – Columns were sequentially equilibrated with 200 µL 95 (v/v)% ACN and 200 µL 150 mM ammonium-formate buffer by centrifugation (1 min, 10,000 rpm), and the flow-through was discarded.
2. Adsorption – Fluorescently labelled glycan samples (180 µL in 85 (v/v)% ACN) were loaded onto the column, incubated for 5 min at room temperature, and centrifuged (1 min, 10,000 rpm). Flow-through fractions were collected; test analyses confirmed no detectable glycan signal.
3. Washing – Columns were washed with 200 µL 95 (v/v)% ACN and centrifuged (1 min, 10,000 rpm).
4. Elution – Glycans were eluted in two steps: first with 50 µL 1 (v/v)% formic acid, then with 50 µL 150 mM ammonium-formate buffer. Each elution was incubated for 3 min prior to centrifugation (1 min, 10,000 rpm). Combined eluates were collected for HILIC-UHPLC-MS analysis.

### Automation of 0.5 mg/ml NiFe_2_O_4_-NH_2_ MNP based N-glycan purification protocol

Automated sample preparation was performed on a Hamilton Microlab Prep liquid handling platform (Hamilton Company Americas, Australia & Pacific Rim) using a robot compatible, 96-well PCR plate (Hamilton Company Americas, Australia & Pacific Rim) and four 15 mL reagent reservoirs containing: (i) 100% (ACN), (ii) 0.5 mg/mL NiFe_2_O_4_-NH_2_ MNP suspension, (iii) 95 (v/v)% ACN wash solution, and (iv) 150 mM ammonium-formate buffer (pH 4.4) for elution. The automated workflow was adapted from the optimized manual protocol, with the only modification being the use of 180 µL instead of 200 µL MNP suspension. The purification consisted of sequential steps of particle conditioning, glycan adsorption from labelled samples, washing with 95 (v/v)% ACN, and elution with a150 mM ammonium-formate (pH 4.4) buffer. The detailed 22-step workflow of the MNP-based *N*-glycan purification protocol is included in Table S7.

### UHPLC-FLD-MS analysis of N-glycans

For method optimization, *N-*glycan samples were analysed using a Waters Acquity UHPLC system controlled by Empower 3 software (Waters Corporation, Milford, MA, USA). Separation was performed on a Waters BEH Glycan Amide column (100 × 2.1 mm, 1.7 μm particle size) operated at 60 °C. Samples (5 µL) dissolved in 75/25 (v/v)% ACN/Milli-Q water were injected and separated using a linear gradient of solvent B (100% ACN) from 72% to 55% over 42 min at a flow rate of 0.4 mL/min. Solvent A was 50 mM ammonium-formate buffer (pH 4.4). Fluorescence detection was applied at excitation and emission wavelengths of 309 nm and 359 nm, respectively, optimized for ProA labelled glycans. This setup provided high sensitivity and reproducible separation of glycans, suitable for comparative evaluation of manual and MNP-based purification protocols.

Mass spectrometric measurements were performed using a Waters G2-XS QTof MS instrument (Waters Corporation, Milford, MA, USA) operating in positive ionization mode. The capillary voltage was set to 2.2 kV. The desolvation temperature was maintained at 120 °C with a gas flow rate of 800 L/h. Mass spectra were acquired over an m/z range of 500–2000.

### Software and data analysis

Chromatographic data acquisition and automated peak integration were performed using Empower 3 software (Waters Corporation, Milford, MA, USA), a modular chromatography data system widely used for UHPLC/UPLC workflows. This platform enabled standardized and reproducible glycan peak processing across manual and MNP-based purification experiments.

For statistical analysis and data visualization, GraphPad Prism (version 10.1.2; GraphPad Software, San Diego, CA, USA) was used. Statistical tests included one-way,- and two-way ANOVA, Kruskal-Wallis and Mann-Whitney tests and normality assessments, performed with a 95% confidence interval and a significance threshold of *p* < 0.05. All statistical analyses were verified for normality assumptions prior to applying ANOVA tests. Multiple comparison corrections were applied where appropriate, as outlined below:

**(i) Optimization of dispersion media and concentration**

- one-way ANOVA with Dunnett’s test comparing the protocols to the benchmark Protocol A.
- Kruskal-Wallis test followed by Dunn’s test to identify significant differences between dispersion media regarding the relative distribution of glycans.

**(ii) Comparative evaluation of elution buffers and commercial kits**

- two-way ANOVA with Tukey’s test (between the protocol with respect for one elution buffer.
- one-way ANOVA with Dunnett’s test comparing the amine-and amide-column kits to the benchmark Protocol C (0.5 mg/mL MNP).
- Kruskal–Wallis test followed by Dunn’s test to identify significant differences between elution buffers and among the different glycan purification protocols regarding the relative distribution of glycans.

**(iii) Comparison of manual and automated sample purification workflows**

- one-way ANOVA followed by Tukey’s multiple comparisons test for the comparison of total glycan intensities.
- Mann-Whitney statistical analysis to identify significant differences between manual and automated workflows regarding glycan’s relative abundances.
- Kruskal-Wallis with Dunn’s test for the comparison of the eight parallel IgG derived samples.

## Supplementary Information

Below is the link to the electronic supplementary material.

Supplementary Material 1

Supplementary Material 2

## Data Availability

The generated data can be requested from the corresponding author.

## Declarations

### Sample availability

Samples of the nanoparticles are available from the authors.
