# Supplementary material for: Automation of N-glycan purification using amine-functionalized NiFe2O4 magnetic nanoparticles
Source: Sci Rep. 2026 Jun 8;16:26094. doi: 10.1038/s41598-026-54879-1 (PMC13490495; doi:10.1038/s41598-026-54879-1)
Supplement: Supplementary file 1 [file 41598_2026_54879_MOESM1_ESM.docx]

Supplementary Material 1

Automation of *N*-Glycan Purification Using Amine-Functionalized NiFe₂O₄ Magnetic Nanoparticles

Dalma Dojcsák^1^*, Ágnes Mária Ilosvai^1,2^, László Vanyorek^2^, Csaba Váradi^2^

^1^	Higher Education and Industrial Cooperation Centre, University of Miskolc, Hungary 3515

^2^	Institute of Chemistry, University of Miskolc, Hungary 3515

*****Correspondence: dalma.dojcsak@uni-miskolc.hu; Tel.: +36-30-355-7442

**Figure S1.** Comparison of MNP-based protocols with two elution buffers and commercial amine-and amide MonoSpin-kits. (A) Ten most abundant relative glycan abundances were illustrated by elution buffers (E1: Milli-Q water, E2: 150 mM ammonium-formate, pH 4.4). (B) Free dye intensity be-tween different MNP-protocols and elution buffers illustrated in logarithm scale. Statistical test: two-way ANOVA with Tukey’s multiple comparison test between the protocols and Bonferroni’s multiple comparison test between the elution buffers. (***)p < 0.001, (****)p < 0.0001 ns, not significant

**Figure S2.** Raw data of free dye intensity among validated 0.5 mg/mL NiFe_2_O_4_-NH_2_ MNP based protocol and commercial kit. Statistical test: one-way ANOVA with Dunnett’s multiple comparison test. (**)p < 0.01.
